# Laser in situ keratomileusis for astigmatism ≤ 0.75 Diopter combined with low myopia: a retrospective data analysis

**DOI:** 10.1186/1471-2415-14-1

**Published:** 2014-01-06

**Authors:** Toam Katz, Andreas Frings, Stephan J Linke, Gisbert Richard, Vasyl Druchkiv, Johannes Steinberg

**Affiliations:** 1 Department of Ophthalmology, University Medical Center Hamburg-Eppendorf, Martinistrasse 52, Hamburg, 20246, Germany

**Keywords:** Alpins vector method, Cylinder axis, Induced astigmatism, Laser in situ keratomileusis, Low astigmatism, Low myopia

## Abstract

**Background:**

This study examined the refractive and visual outcome of wavefront-optimized laser in situ keratomileusis (LASIK) in eyes with low myopia and compound myopic astigmatism ≤ 0.75 diopter (D).

**Methods:**

153 eyes from 153 consecutive myopic patients (74 male, 79 female; mean age at surgery 40.4 ± 10.4 years) who had a preoperative refractive cylinder ≤ 0.75 D and a manifest sphere between -0.25 D and -2.75 D, and who had completed 4-month follow-up. Three subgroups defined by the magnitude of preoperative manifest refractive cylinder (0.25, 0.50, and 0.75 D) were formed. Manifest refraction, uncorrected and corrected visual acuity were assessed pre- and postoperatively. The astigmatic changes achieved were determined using the Alpins vector analysis.

**Results:**

After 4 months (120.0 ± 27.6 days) of follow-up, a mean uncorrected distant visual acuity of 0.07 ± 0.11 logMAR and a mean manifest refraction spherical equivalent of -0.06 ± 0.56 D were found. There was no statistically significant difference in efficacy and safety between the preoperative cylinder groups. Astigmatic overcorrection for preoperative cylinder of ≤ 0.50 D was suggested by the correction index, the magnitude of error, the index of success, and the flattening index.

**Conclusions:**

Low myopic eyes with a preoperative cylinder of ≤ 0.50 D were significantly overcorrected with regard to cylinder correction when combined with low myopic LASIK. Accordingly, we are cautious in recommending full astigmatic correction for eyes with low myopia and manifest cylinder of ≤ 0.50 D.

## Background

Over the last two decades, the field of laser refractive surgery has seen dramatic advances introduced by new technologies [1]. Laser in situ keratomileusis (LASIK) has become the most popular excimer technique among refractive surgeons as it provides numerous major clinical and refractive benefits [2].

Normally, a refractive procedure aims at achieving a plano refractive result. However, slight inaccuracy in the reduction and even the induction of an astigmatic error has been reported, and these can limit uncorrected distance visual acuity (UDVA). This results in a frustrating situation as uncorrected or induced astigmatism, even as low as 1.00 diopter (D), has been identified as a cause of significantly decreased vision [3].

Recently, we showed that full astigmatic correction of low myopic astigmatism tended to result in astigmatic overcorrection [4], and that this was especially evident in eyes with a preoperative refractive cylinder of ≤ 0.50 D. However, the focus of that analysis was on eyes with only moderate to high myopia. Moreover, we did not analyze whether the axis of the preoperative cylinder could be a possible predictor of post-LASIK refractive outcome. This may be of importance as a recent study found that eyes with oblique astigmatism (OBL) had significantly lower visual performance [5].

The current investigation therefore examined the refractive and visual outcome after wavefront-optimized LASIK in eyes with low myopia between -0.25 and -2.75 D and astigmatism of ≤ 0.75 D. We also assessed the effect of cylindrical axis orientation and its impact on refractive results.

## Methods

### Patients

This study examined 153 eyes from 153 consecutive myopic patients with preoperative manifest sphere between -0.25 and -2.75 D, and refractive cylinder of ≤ 0.75 D and who had completed a 4-month follow-up. We randomly selected one eye from each patient for analysis. Exclusion criteria were ocular pathology, and/or medication likely to influence the stability of refractive error.

All patients were treated between 2006 and 2010. All data is based on the Hamburg Refractive Data Base (Data retrieved from Care Vision Germany). Informed consent for retrospective data analysis was obtained from refractive surgery candidates after explanation of the nature and possible consequences of the study and approval of local ethics committee (no. 2882) was achieved. Our study adhered to the tenets of the Declaration of Helsinki and was approved by the local ethics committee.

Patients were assigned to one of three groups (0.25, 0.50, 0.75 D) according to the magnitude of their preoperative refractive manifest cylinder. Manifest spherical and cylindrical refractions, and visual acuity with and without correction were assessed before and 1 day, and 1 and 4 months after operation, and recorded electronically. However, all results reported here are based on the data from the 4-month (120.0 ± 27.6 days) follow-up (see Table 1). The spherical and cylindrical refractions were acquired by subjective refraction. The refractive outcome was analyzed according to standard graphs for reporting the efficacy, predictability, and safety of refractive surgery, as suggested by Dupps [6]. The results are based on first treatments only; enhancements were not included.

**Table 1:** Epidemiological data and refractive parameters. Displayed as aggregate data for each of the cylinder groups

	**Cylinder 0.25**				**Cylinder 0.50**				**Cylinder 0.75**				**Total**			
	**Number**		**%**		**Number**		**%**		**Number**		**%**		**Number**		**%**	
**Eyes**	37		24,2		81		52,9		35		22,9		153		100	
**Male**	17		45,95		39		48,15		18		51,43		74		48,37	
**Female**	20		54,05		42		51,85		17		48,57		79		51,63	
**Patients**	37		100		81		100		35		100		153		100	
	**Range**		**Mean(±SD)**		**Range**		**Mean(±SD)**		**Range**		**Mean(±SD)**		**Range**		**Mean(±SD)**	
**Age (years)†**	23/60		38.84(10.56)		20/61		39.68(10.65)		24/63		43.49(9.28)		20/63		40.35(10.42)	
**Days after OP‡**	70/168		121.47(23.13)		62/169		121.75(28.8)		63/166		114.32(29.13)		62/169		120.01(27.63)	
	**PreOP**		**PostOP**		**PreOP**		**PostOP**		**PreOP**		**PostOP**		**PreOP**		**PostOP**	
**SE**	-2.88/-0.63	-2(0.7)	-1/1	0.01 (0.42)	-3/-0.5	-2.27 (0.68)	-1.5/1	-0.06 (0.59)	-2.88/-0.63	-1.96 (0.74)	-1.25/1.13	-0.16 (0.61)	-3/-0.5	-2.13 (0.71)	-1.5/1.13	-0.06 (0.56)
**Subje ctiveS ph**	-2.75/-0.5	-1.88 (0.7)	-0.75/1.25	0.29 (0.42)	-2.75/-0.25	-2.02 (0.68)	-1.25/1.5	0.27 (0.66)	-2.5/-0.25	-1.59 (0.74)	-1/1.75	0.16 (0.63)	-2.75/-0.25	-1.88 (0.72)	-1.25/1.75	0.25 (0.6)
**Subje ctiveC yl**	-0.25/-0.25	-0.25 (0)	-1.25/0	-0.55 (0.29)	-0.5/-0.5	-0.5(0)	-1.75/0	-0.66 (0.4)	-0.75/-0.75	-0.75 (0)	-1.5/0	-0.65 (0.41)	-0.75/-0.25	-0.5(0.17)	-1.75/0	-0.63 (0.38)
**UDVA LogM**	0.1/2	0.78 (0.3)	-0.11	0.05 (0.1)	0.1/2	0.84 (0.3)	-0.18	0.08 (0.1)	0.1/2	0.75 (0.4)	-0.18	0.07 (0.1)	0.1/2	0.81 (0.3)	-0.18	0.07 (0.1)

†The difference in age was not significant, F(2,152) = 2.178, p = 0.117.

‡ The difference in follow up time was not significant, K-W (2) = 1.9, p = 0.387.

### Surgical technique

The LASIK procedure included mechanical flap preparation using an automated microkeratome (M2, Moria, France) with a single-use 90-μm head. For all eyes, the excimer ablation was performed with an Allegretto excimer laser platform (Eye-Q 200 Hertz (Hz) or 400 Hz, WaveLight GmbH, Erlangen, Germany) under constant eye tracking (250 Hz). To minimize the induced spherical high-order aberration, an aspherical “wavefront-optimized” profile was used with an optical zone of 6.0, 6.5 or 7.0 mm depending on the mesopic pupil diameter and expected residual stromal bed [7]. The manufacturer-recommended “WaveLight myopic astigmatic nomogram” was implemented to compensate for very short or long ablation time and for a cylinder-sphere coupling effect. However, there was no coupling effect in cylinder between 0 and -3 D, but the laser produces compensation for ablation time for sphere from -0.25 to -2.00 D.

Cyclotorsion was minimized using a “NeuroTrack” system (WaveLight GmbH) in which four built-in blinking light sources eliminate cyclotorsion at its source by controlling optokinesis.

Since the level of dark adaptation is extremely difficult to standardize between subjects, all subjects were asked to wait in an area of dim illumination for 2 minutes prior to examination. Mesopic pupil size was determined with the Colvard pupillometer with surrounding background room illumination of approximately 0.15 lux as measured with a luxmeter by well trained optometrists. The handheld Colvard pupillometer uses light amplification technology. The patient is asked to fixate on a red light produced by an infrared LED inside the device and the examiner is able to focus the iris and pupil by moving the pupillometer slightly forward and backward. A millimetre ruler is superimposed by a reticule in the device over the image and allows direct measurement. The examiner was instructed to estimate the size of the horizontal pupil diameter to within 0.25 mm. The other eye was covered by the patients hand during the measurement.

The laser treatments were performed in nine Care Vision Refractive Centers located in Berlin, Cologne, Frankfurt/ Main, Hamburg, Hanover, Munich, Nuremberg, Stuttgart, and Vienna by 23 experienced refractive surgeons. All surgeons followed a standard protocol of indications, preoperative, intraoperative and postoperative management written by the first author (T.K.), and were trained by him in situ. Postoperative medication for LASIK included ofloxacin four times a day for 1 week, and dexamethasone four times a day for the 1st week, and two times a day for the 2nd and 3rd weeks. Preservative-free hyaluronic acid artificial tears (Hylolasop, Ursapharm GmbH, Germany) were applied to all eyes for 1–3 months.

### Vector analysis by the Alpins vector method

The Alpins vector method was used to assess the refractive effects of astigmatic correction [8]. Clinical notations of pre- and postoperative cylinder power and cylinder axis were converted to a target induced astigmatism (TIA) vector, a surgically induced astigmatism (SIA) vector, and a difference vector (DV) [9]. For each of the three cylinder groups, the index of success (IOS), the flattening index (FI), the correction index (CI), the magnitude of error (ME), and the angle of error (AE) were calculated as combined aggregate data [10]. These indices clarify whether the treatment was on- or off-axis, whether too much or too little treatment was applied, and the theoretical amount and orientation of the astigmatism treatment required to achieve the targeted plano cylindrical outcome [10]. Calculations for the Alpins vector analysis were performed using Microsoft Office Excel Software (MS Office version 2007; Microsoft, Corp., Redmond, WA). Cylinder refractive data in demographics is in Minus but analyzed in Alpins’ vector analysis in + sign. It does not change the vector analysis.

### Analysis of cylinder axis

To assess whether the preoperative cylinder axis had any influence on the efficacy, safety or predictability of the LASIK treatment, subgroups were defined according to the preoperative cylinder axis (with-the-rule (WTR), axis 0 ± 22.5°; against-the-rule (ATR), axis 90 ± 22.5°; or oblique (OBL), axis 45 ± 22.5°). For each subgroup, we examined whether there was a significant change between the pre- and postoperative axis, and, if there was, whether one type of axis was more likely to change. We also investigated whether eyes with a change of cylinder axis had poorer refractive results compared to those without.

### Statistical analysis

Once the data were compiled, they were entered into a spreadsheet program (Microsoft Office Excel; Hamburg Refractive Data Base) and were statistically analyzed using predictive analytical software (SPSS version 17.0; SPSS, Inc., Chicago, IL). For statistical analysis, data description was based on medians and quartiles of the respective calculations. All data, except age, were analyzed with non- parametric methods. A Kruskal–Wallis or Mann–Whitney test was used for comparisons of continuous measures between groups. A value of P <0.05 was considered statistically significant. Predictability was examined with an ordinary least squares regression analysis.

## Results

Epidemiological data, pre- and postoperative manifest spherical and cylindrical refractions, corrected distant visual acuity (CDVA) and UDVA are summarized in Table 1. We had 143 eyes in “post-op 1” (20–59 days), 153 eyes in “post-op 2” (60–170 days) and 143 eyes in both intervals. The dropout rate is thus 10 eyes from 153, i.e. 6.5%. To rule out systematic differences between 200 Hz and 400 Hz lasers, and thus between our centers, a Kruskal–Wallis test was applied; no systematic differences were found (see Table 2).

**Table 2:** Efficacy (EI) and safety (SI), displayed by preoperative astigmatic cylinder magnitude

**Cylinder (D)**	**-0,25**	**-0,5**	**-0,75**	p-Value**	**Total**
**N total (Eyes/Patients)**	37	81	35	*-*	153
EI total*	0.93(0.68-1)	0.75(0.67-0.94)	0.8(0.67-1)	0,211	0.78(0.67-0.92)
SI total*	1(1–1.08)	1(0.92-1.09)	1.05(0.9-1.2)	0,659	1(0.94-1.11)
**N (Eyes/Patients)400Hz**	9	15	6	*-*	30
EI 400Hz*	0.82(0.65-0.94)	0.89(0.78-1)	0.83(0.73-0.98)	*-*	0.81(0.67-0.93)
SI 400Hz*	0.91(0.83-1.03)	1.04(1–1.1)	1.04(0.99-1.22)	*-*	1(0.9-1.07)
**N (Eyes/Patients)200Hz**	28	66	29	*-*	123
EI 200Hz*	0.93(0.73-1)	0.75(0.67-0.94)	0.8(0.65-1)	*-*	0.76(0.66-1)
SI 200Hz*	1(1–1.08)	1(0.92-1.09)	1.05(0.9-1.2)	*-*	1(0.92-1.1)

* Median(Q25-Q75) Q25 = 25^th^ quartile; Q75 = 75^th^ quartile.

**Tested with Kruskal-Wallis Test.

### Predictability

**Figure 1:**
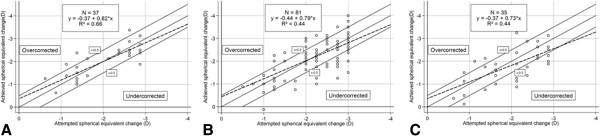
Attempted versus achieved spherical equivalent (SE) correction at 4 months, displayed by preoperative astigmatic cylinder magnitude: A) 0.25 D, B) 0.50 D, and C) 0.75 D.

For the mean follow-up of 4 months (120.0 ± 27.6 days), the mean refractive spherical equivalent (MRSE) decreased from -2.13 ± 0.71 D preoperatively (range – 3.00 to -0.50 D) to -0.06 ± 0.56 D (range -1.50 to 1.13 D), and the mean subjective sphere decreased from -1.88 ± 0.72 D (range -2.75 to -0.25 D) to 0.25 ±0.60 D (range -1.25 to 1.75 D) (see Table 1 and Figure 1). For 102 patients (66.7%), the results were within ± 0.50 D of the attempted correction. The postoperative magnitude of mean manifest cylinder increased in eyes with a preoperative refractive cylinder of ≤ 0.50 D. This resulted in a mean postoperative refractive cylinder of - 0.66 ± 0.40 D (range -1.75 to 0.00 D) in eyes with a preoperative refractive cylinder of 0.50 D, and a mean postoperative cylinder of -0.55 ± 0.29 D (range -1.25 to 0.00 D) in eyes of the 0.25 D group (see Tables 1 and 3). Of the 118 eyes with a preoperative refractive cylinder of ≤ 0.50 D, 30 (25.4%) had a postoperative astigmatism of ≥ 1.00 D. In eyes with preoperative astigmatism of 0.75 D, the refractive cylinder decreased to a mean value of -0.65 ± 0.41 D (range -1.50 to 0.00 D) postoperatively.

**Table 3:** Predictability of spherical equivalent displayed by preoperative astigmatic cylinder magnitude

	Predictability of Spherical Equivalent*					
	**under correction <1.0**	**1.0 to 0.51**	**±0.50**	**0.51 to 1.0**	**over correction >1.0**	**Total**
	**-0,25 D**					
**Number**	0	1	31	5	0	37
*%*	*0*	*3*	*84*	*14*	*0*	*100*
	**-0,50 D**					
**Number**	4	12	52	13	0	81
*%*	*5*	*15*	*64*	*16*	*0*	*100*
	**-0,75 D**					
**Number**	1	10	19	4	1	35
*%*	*3*	*29*	*54*	*11*	*3*	*100*

*Exact significance, Fisher’s Exact Test = 0.027.

### Efficacy

**Figure 2:**
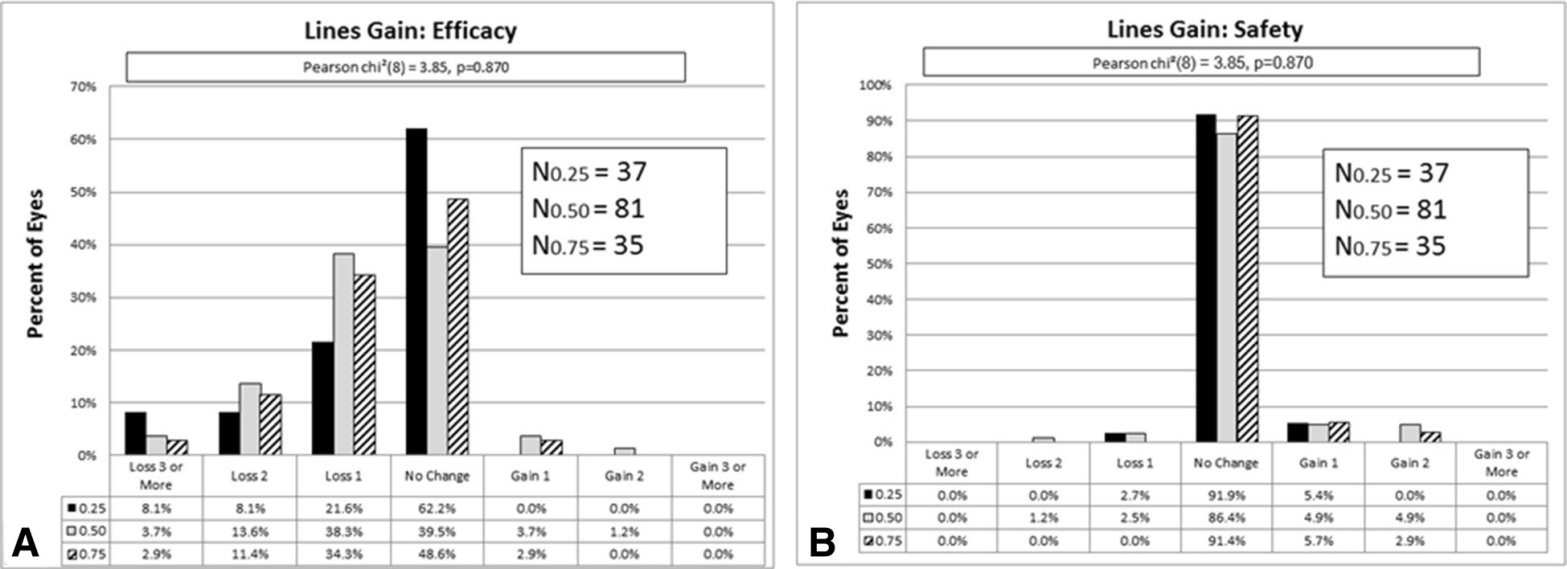
Change in lines of A) efficacy, and B) safety (preoperatively vs. 4 months postoperatively).

**Figure 3:**
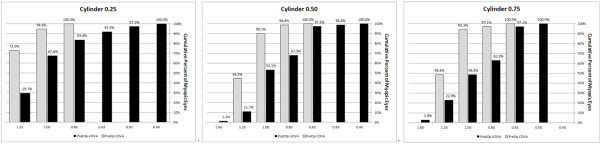
Preoperative CDVA versus UDVA 4 months postoperatively.

The median overall efficacy was 0.78. There was no statistically significant difference in efficacy (P = 0.211, Mann–Whitney test) between the preoperative cylinder groups (see Table 2). Postoperative UDVA was equivalent to preoperative CDVA in 50.1% of all eyes (see Figures 2 and 3). A loss of three or more lines in efficacy was obtained for 4.9%.

### Safety

The mean preoperative CDVA for all eyes was -0.03 ± 0.06 logMAR (range -0.08 to 0.17 logMAR). At the 4-month follow-up, the mean CDVA was -0.04 ± 0.07 logMAR (range -0.20 to 0.22 logMAR) (see Table 1). There was no statistically significant difference in safety between the preoperative cylinder groups (P = 0.659, Mann- Whitney test). Each group had a mean safety value of ≥ 1.0. Independent of preoperative refractive cylinder, 89.9% of all eyes did not change lines in safety; 5.3% gained one line postoperatively (see Figures 2 and 3). No eyes had vision- threatening complications.

### Analysis of cylinder axis

Although there were no significant differences between preoperative cylinder subgroups (see Table 4), the number of cases with preoperative ATR astigmatism decreased from 45 to 12 (decrease of 73%), whereas eyes with WTR astigmatism increased from 50 to 86 (increase of 72%). Efficacy and safety indicate that there was no significant difference in refractive results. Predictability of spherical equivalent (SE) was high in all cylinder axis groups as 96% of all eyes were within ± 1.00 D of the achieved SE.

**Table 4:** Cylinder axis preop. vs. postop, number of cases with axis change after treatment

	**PreOp§**		
	**-0.25 (n = 41)**	**-0.50 (n = 84)**	**-0.75 (n = 38)**
**WTR (n = 50)**	12 (24%)	26 (52%)	12 (24%)
**Oblique (n = 58)**	17 (29%)	30 (52%)	11 (19%)
**ATR (n = 45)**	8 (18%)	25 (56%)	12 (27%)
	**PostOp¶**		
**WTR (n = 86)**	20 (24%)	44 (51%)	22 (26%)
**Oblique (n = 55)**	13 (24%)	30 (55%)	12 (22%)
**ATR (n = 12)**	4 (33.3%)	7 (58.3%)	1 (8.3%)
	**Axis change‡**		
	**PostOp**		
**PreOp**	WTR (n = 86)	Oblique (n = 55)	ATR (n = 12)
**WTR (n = 50)**	27 (54%)	18 (36%)	5 (10%)
**Oblique (n = 58)**	33 (57%)	21 (36%)	4 (7%)
**ATR (n = 45)**	26 (57.8%)	16 (35.6%)	3 (6.7%)

§ Not significant, χ^2^(4) = 2.18, p = 0.704.

Not significant, Fisher’s Exact Test, p = 0.756.

‡ Not significant, Fisher’s Exact Test, p = 0.976.

### Vector analysis

As expected, the amount of astigmatic change induced by surgery (SIA) was statistically significant different between the preoperative cylinder subgroups (P = 0.001). This was confirmed by a median DV of 0.50 D (range 0.25 – 1.00 D) (P = 0.423) (see Table 5).

**Table 5:** Vectors and indices examined by the Alpins method: Data

**Cylinder**	**0.25 D**		**0.50 D**		**0.75 D**		**Total**		**P**
**Component**	**Min-Max**	**Median**	**Min-Max**	**Median**	**Min-Max**	**Median**	**Min-Max**	**Median**	
		**(Q25/Q75)**		**(Q25-Q75)**		**(Q25-Q75)**		**(Q25-Q75)**	
*TIA Magnitude*	0.25 /0.25	0.25(0.25/0.25)	0.50 / 0.50	0.50(0.50/0.50)	0.75 / 0.75	0.75(0.75/0.75)	0.25 / 0.75	0.50(0.38/0.50)	*0.000*
*Angle*	3 / 180	87(53/142.5)	3 / 180	80(50/132.75)	3 / 177	94(29/121.5)	3 / 180	86(50/131.5)	*0.872*
*SIA Magnitude*	0.05 /3.20	0.64(0.33/0.83)	0.02 / 2.14	0.91(0.61/1.24)	0.21 / 1.92	0.99(0.61/1.25)	0.02 / 3.20	0.82(0.54/1.23)	*0.001*
*Angle*	19 / 172	82(62.2/109.09)	4 / 177	82(57.66/113.36)	5 / 173	96(68.44/122.09)	4 / 177	86(61.3/113.84)	*0.350*
*DV Magnitude*	0.00 /3.00	0.50(0.31/0.94)	0.00 / 1.75	0.50(0.25/1.00)	0.00 / 1.50	0.50(0.50/1.00)	0.00 / 3.00	0.50(0.50/1.00)	*0.839*
*Angle*	0 / 180	138.5(21.25/166.75)	0 / 180	122.5(21.25/164)	0 / 180	63(22.5/163.5)	0 / 180	125(21.5/164.5)	*0.722*
Correction index	0.21 /12.78	2.57(1.3/3.31)	0.03 / 4.28	1.82(1.23/2.49)	0.28 / 2.56	1.32(0.82/1.67)	0.03 /12.78	1.77(1.11/2.51)	*0.000*
Magnitude of error	-0.2 / 2.95	0.39(0.08/0.58)	-0.48 / 1.64	0.41(0.11/0.74)	-0.54 / 1.17	0.24(-0.14/0.5)	-0.54 / 2.95	0.37(0.04/0.66)	*0.079*
Angle of error	-119.26 /146	-2.08(-44.7/30.49)	-175.8 /142.45	0.5(-11.47/13.82)	-69.32 /159.39	0(-11.92/12.96)	-175.8 /159.39	0(-15.53/14.66)	*0.565*
Index of success	0 / 12	2(1.25/3.75)	0 / 3.5	1(0.5/2)	0 / 2	0.67(0.67/1.33)	0 / 12	1(0.67/2)	*0.000*
Flattening Index	0.02 /12.57	1.41(0.7/2.72)	0.03 / 4.24	1.11(0.52/1.78)	0.02 / 2.49	0.87(0.48/1.15)	0.02 /12.57	1(0.55/1.82)	*0.023*

* Tested with non-parametric Kruskal-Wallis Test.

The CI differed significantly (P < 0.001) between the preoperative refractive cylinder groups. The median CI value of 1.66 indicated that an overcorrection was achieved, which was especially evident in eyes with preoperative cylinder of ≤ 0.50 D (see Table 5).

The ME showed borderline significance (P = 0.047) between the preoperative cylinder subgroups. Its distribution indicated that especially in eyes with preoperative refractive cylinder of 0.50 D a cylindrical overcorrection was induced by surgery, thereby confirming the CI results. For all preoperative cylinder magnitudes, a median AE of zero or close to, indicated an on-axis treatment.

The IOS differed significantly (P < 0.001) between the preoperative cylinder groups (see Table 5). Therefore, by definition, the treatment in eyes with preoperative refractive cylinder of ≤ 0.50 D was less accurate.

The FI revealed borderline insignificance between the preoperative cylinder groups (P = 0.09). Eyes with a preoperative cylinder of ≤ 0.50 D achieved a median FI of 1.13 (range 0.47–2.47), which is a parameter of too strong flattening induced by surgery (see Table 5) thereby confirming the overcorrection revealed by the CI and ME.

## Discussion

The current study explores the outcome of LASIK in eyes with low and compound low astigmatism, focusing on the effectiveness of full astigmatic component correction and the role of the preoperative cylinder axis.

Wolffsohn [3] recently showed that uncorrected astigmatism, even as low as ≤ 1.0 D, causes significantly decreased vision. We [4] were the first to assess the effectiveness of the astigmatic component correction in eyes with a low preoperative refractive cylinder. The current study confirms a tendency for low astigmatism in eyes with preoperative refractive cylinder of ≤ 0.50 D to be significantly overcorrected. Previous studies have generally supported the use of LASIK for the treatment of low myopia. Balaszi [11] reported that, independent of the preoperative sphere, the safety, efficacy, and predictability were similar when using LASIK with a scanning excimer laser. Van Gelder [12] compared patients treated with photorefractive keratectomy (PRK) and LASIK. The authors concluded that for low myopia both methods yielded a comparable visual and refractive outcome. Most LASIK studies of low myopic eyes have reported that more than 90% of eyes achieved a CDVA of 6/12 or better [13,14]. In those studies, postoperative MRSE within ±1.00 D was achieved for more than 90% of eyes. Our results compare well with those as we obtained a mean UDVA of 0.07 ± 0.11 logMAR and MRSE of -0.06 ± 0.56 D at the 4-month follow-up. Postoperative MRSE within ± 1.00 D was achieved by more than 96.1% (n = 147) of eyes.

Although nine medical centers were involved, all eyes of the current study were treated according to the same surgical protocol, our data compare favorably with those reported previously [4] as undesired coupling effects were efficiently counteracted by the WaveLight nomogram recommended by the laser platform manufacturer. There are no systematic differences in results between centers using 200 Hz and 400 Hz lasers [4]. The low predictability of SE (64.67%) relative to the small cylinder correction, and thus low efficacy levels reported here, are based on the inclusion of only patients who had completed a 3-month follow-up. Although our patients were invited for a complete follow-up, they were not obliged to attend. Those with a very good postoperative UDVA of logMAR 0.0 or better in the early postoperative exams tended not to show up for the full 3-month follow-up, whereas those with lower UDVA were motivated to be rechecked and retreated (as mentioned in methods, we only included the results of the first treatment). These non-attendees are probably the reason for the negative bias of the efficacy and the sub-standard effectivity. We see this as a drawback in our analysis.

Although a mean UDVA of 0.09 ± 0.14 logMAR and MRSE of -0.02 ± 0.68 D were obtained at the 4-month follow-up, conflicting results are suggested by the vector analysis (see Table 5). Full astigmatic correction of low myopic astigmatism tends to result in astigmatic overcorrection and, therefore, the amount of planned cylinder correction should be carefully considered. This is confirmed by total DV with a median magnitude of 0.50 D (range 0.00–3.00 D).

Whenever a new axis of the cylinder is surgically induced, astigmatic changes occur. In eyes with regular astigmatism, the axis of the cylinder may be WTR, ATR or OBL. Kobashi [5] demonstrated that eyes with OBL astigmatism have significantly lower visual performance, which led us to speculate whether post-LASIK refractive results depend on the type of preoperative astigmatic axis. Our data show that there were no statistically significant differences in safety and efficacy (see Tables 6, 7, 8 and 9), between eyes with different astigmatic axis or in eyes with axis change after LASIK.

**Table 6:** Efficacy (EI) and safety (SI) predictability

**Axis (D)**	**WTR**	**Oblique**	**ATR**	p-Value**
N total (Eyes/Patients)	50	58	45	*-*
EI Total*	0.80(0.67-1.0)	0.75(0.67-1.0)	0.82(0.70-1.0)	0,414
SI Total*	1(0.92-1.10)	1(0.92-1.10)	1.0(0.93-1.10)	0,985

* Median(Q25-Q75) Q25 = 25^th^ quartile; Q75 = 75^th^ quartile.

**Tested with Kruskal-Wallis Test.

**Table 7:** Predictability, displayed by preoperative cylinder axis

	Predictability of Spherical Equivalent*					
	**undercorrection <1.0**	**1.0 to 0.51**	**±0.5**	**0.51 to 1.0**	**overcorrection >1.0**	**Total**
	**WTR**					
**Number**	1	9	34	6	0	50
*%*	*2*	*18*	*68*	*12*	*0*	*100*
	**Oblique**					
**Number**	3	8	38	9	0	58
*%*	*5*	*14*	*66*	*16*	*0*	*100*
	**ATR**					
**Number**	1	6	30	7	1	45
*%*	*2*	*13*	*67*	*16*	*2*	*100*

*Exact significance, Fisher’s Exact Test = 0.940.

**Table 8:** Efficacy (EI), and safety (SI) predictability

**Change of the axis**			
**Axis (D)**	**No change**	**Change**	p-Value**
N total (Eyes/Patients)	51	102	*-*
EI total*	0.82(0.67-1.0)	0.78(0.67-1.0)	0,823
SI total*	1.0(0.91-1.13)	1.0(0.98-1.09)	0,762

**Table 9:** Predictability, displayed by axis change after treatment

	Predictability of spherical equivalent*					
	**under correction <1.0**	**1.0 to 0.51**	**±0.5**	**0.51 to 1.0**	**over correction >1.0**	**Total**
	**No change**					
**Number**	2	6	38	5	0	51
*%*	*4*	*12*	*75*	*10*	*0*	*100*
	**change**					
**Number**	3	17	64	17	1	102
*%*	*3*	*17*	*63*	*17*	*1*	*100*

*Exact significance, Fisher’s Exact Test = 0.589.

However, efficacy was lower in eyes with preoperative OBL astigmatism. The number of eyes with ATR astigmatism decreased by 73% after surgery, whereas cases of WTR astigmatism increased by 71%. As the number of eyes with OBL astigmatism did not change, our data suggest that most eyes with preoperative ATR had WTR astigmatism after LASIK. This is supported by Huang [14], who mentioned an average of 0.12 D “WTR” astigmatism induced after surgery, with the flap creation thought to be the strongest determinant for postoperative astigmatism. Creating the corneal flap is one of the crucial steps within a LASIK surgery. In fact, previous studies have examined that the lamellar cut made by the microkeratome can modify the existing refractive error thereby inducing astigmatism which may limit UDVA or consequence in subjective symptoms such as halos and night vision problems. The applied mikrokeratome uses a superior hinge position. The role of different hinge position on the outcome of LASIK has widely been discussed. Huang et al. [14] reported that there is an average of 0.12 D “with the rule” astigmatism induced after surgery with the flap creation being attributed as strongest determinant on postoperative astigmatism. Lee et al. [15] compared patients undergoing LASIK assigned to superior and nasal hinges and find no difference in flap complication rates or visual outcomes. This is supported by Guell [16] who report that there is no statistical difference with varying hinge position using simulated keratometry. On the other hand Pallikaris et al. [17] demonstrate that horizontal coma increases after using microkeratomes that create nasal hinges.

In eyes with a preoperative refractive cylinder ≤ 0.50 D, the mean subjective cylinder was increased at 4-month follow-up (see Table 1). This finding is important when discussing residual or induced astigmatism after LASIK as the cause of decreased UDVA or monocular diplopia [2,3]. Especially eyes of the 0.25 D group were characterized by relatively considerable overcorrection of the preoperative refractive cylinder, as shown by the IOS. A full correction of astigmatism is achieved when SIA equals TIA in magnitude and angle [18]. The ME was statistically significant (P = 0.047) higher for eyes with refractive cylinder of ≤ 0.50 D compared to those with 0.75 D, resulting in an overcorrected cylinder in the preoperative ≤ 0.50 D cylinder subgroup. Our analysis indicates that too much flattening was applied in eyes with a preoperative refractive cylinder of ≤ 0.50 D (P = 0.09). This is especially evident in eyes with a preoperative refractive cylinder of 0.25 D, which is further evidenced by a median CI of 2.13, which strongly indicates astigmatic overcorrection. Moreover, the IOS was significantly (P < 0.001) elevated in eyes with a preoperative refractive cylinder of ≤ 0.50 D, confirming astigmatic overcorrection. The current data contribute to the notion that this overcorrection is uniform over the entire spherical range, as our results confirm our recently published study [4] in which we did not find any statistically significant differences in efficacy and safety between eyes with moderate and high amounts of preoperative sphere.

Our data are based on first treatments only (enhancements are not included), and it was calculated based on the data from only the 4-month follow-up. However, we think the “medium” follow-up is correct for our clinical question and does not have to be extended to longer follow-up. Our standard protocol includes follow-up examinations after 1,7,30 days and 3 to 6 months after which the patients are discharged. Those who show up later than 6 months are much fewer and strongly biased to worse results and need for enhancement. A longer follow-up would have a much higher drop-out and worse effectivity and predictability, especially in eyes with very low myopic astigmatism. Moreover, LASIK flaps and wound healing are usually stable after a few weeks. There was no difference in effectivity between 20–60 days and 50–170 days (data not published). We assume that after 4 months new changes do not necessarily arise from the LASIK nomogram, flap properties or healing process but from a subjective and unpredictable progressive myopisation or change of accommodation.

The safety values of the current study are comparable to previously reported data. However, there are several limitations to our study. First, the number of subjects was small, and thus statistical analysis was limited. Second, a further parameter to assess in future studies is the optical zone treatment diameter, which may play a more important role in the treatment of lower refractive errors as its relative contribution to surgical ablation may have a higher impact. Another bias might have been introduced by the fact that all the presented astigmatic values were based on manifest refraction, with the effect of topographic astigmatism not being explored. Another drawback is that subjective symptoms such as halos and night vision problems, which might be related to postoperative astigmatism, were not systematically recorded. Last, but not least, different hinge positions (superior versus nasal) and their effect on SIA require further study on the effectiveness of correcting low amounts of astigmatism, as current results were based on a superior hinge position only.

## Conclusions

To conclude, our results indicate that low myopic eyes with a preoperative refractive cylinder of ≤ 0.50 D treated with our setup and according to the manufacturer’s nomogram were significantly overcorrected. This corroborates to the notion that overcorrection of low magnitudes of cylinder is independent of the degree of spherical ametropia. Based on the data presented here, we are cautious about treating full refractive cylinders of ≤ 0.50 D using wavefront-optimized LASIK. However, our results are influenced by selection bias, and are laser specific; other laser platforms may yield different results. We did not obtain any statistically significant differences in outcome measurements for different preoperative cylinder axes.

To assess the treatment of very low astigmatism and evaluate existing nomograms, further investigations should be initiated evaluating factors that induce small amounts of astigmatism.

## Abbreviations

LASIK: Laser in situ keratomileusis; UDVA: Uncorrected distance visual acuity; OBL: Oblique astigmatism; Hz: Hertz; TIA: Target induced astigmatism; DV: Difference vector; IOS: Index of success; FI: Flattening index; CI: The correction index; ME: The magnitude of error; AE: The angle of error; WTR: With-the-rule; ATR: Against-the-rule; CDVA: Corrected distant visual acuity; MRSE: Mean refractive spherical equivalent; SE: Spherical equivalent; PRK: Photorefractive keratectomy.

## Competing interests

The authors declare that they have no competing interests.

## Authors’ contributions

TK and AF: have made substantial contributions to conception and design, acquisition of data, analysis and interpretation of data. JS have been involved in drafting the manuscript or revising it critically for important intellectual content. GR: has been involved in revising the manuscript critically and has given final approval of the version to be published. VD: have been involved in drafting the manuscript or revising it critically for important intellectual content. SL: has been involved in revising the manuscript critically and has given final approval of the version to be published. All authors read and approved the final manuscript.

## Pre-publication history

The pre-publication history for this paper can be accessed here:

http://www.biomedcentral.com/1471-2415/14/1/prepub
